# 523. Skin Cell Suspension Autograft Reduces Length of Stay in Deep Partial-thickness Burns

**DOI:** 10.1093/jbcr/irag033.220

**Published:** 2026-04-06

**Authors:** Jonathan E Schoen, M Victoria P Miles, Jeffrey E Carter, Bart D Phillips

**Affiliations:** UCI Health Regional Burn Center, New Orleans, LA; University Medical Center Burn Center, Louisiana State University Health Sciences Center, Department of Surgery, New Orleans, New Orleans, LA; University Medical Center Burn Center, Louisiana State University Health Sciences Center New Orleans, New Orleans, LA; BData Inc, Minneapolis, MN

## Abstract

**Introduction:**

Skin cell suspension autograft (SCSA) is an alternative to split-thickness skin grafting (STSG) for deep second-degree burns, reducing donor site size and delivering melanocytes for repigmentation. While these advantages are established, the independent effect of SCSA on hospital length of stay (LOS) has not been well defined in this population. The American Burn Association’s Burn Care Quality Program (BCQP) is the largest burn registry and denotes usage of SCSA technology in the database. Our study utilized BCQP to assess the impact of SCSA on LOS in adults with deep second-degree burns treated with STSG or SCSA.

**Methods:**

The BCQP was queried for adults (≥18 years) with second-degree burns treated with STSG or SCSA between January 2019 and December 2024. Patients were excluded for non-burn trauma, death or hospice discharge, or LOS outliers (above mean + 2 SD). Cases were matched 2:1 (STSG: SCSA) by sex, age, total body surface area (TBSA), and inhalation injury, within deciles of TBSA (< 30%). Demographics, LOS, and discharge disposition were collected. Welch’s t-test or Pearson’s chi-square test were used for comparisons. Patient throughput was estimated from LOS differences, and cost savings were derived from published average burn bed cost ($7554/day).

**Results:**

A total of 741 patients (494 STSG: 247 SCSA) were analyzed. Groups were similar in age (mean 45 years), TBSA (10%), and demographics. Treatment with SCSA significantly reduced LOS by an average of 5.61 days (36%) versus STSG, with consistent reductions across TBSA deciles (*p*<.05) (Fig. 1). This translated to capacity for 13 additional patients per bed annually and an estimated $42 377 in cost savings per patient. More SCSA patients were discharged home under self-care (83% vs. 70%), with fewer discharged to other facilities (6% vs. 14%) (*p*=.002).

**Conclusions:**

Treatment with SCSA significantly reduced LOS across deep partial-thickness burns up to 30% TBSA with improved discharge to home, and the potential to deliver increased patient throughput and substantial cost savings compared to STSG.

**Applicability of Research to Practice:**

Integrating SCSA into burn care pathways may reduce hospital burden, enhance patient recovery, and increase system capacity. By simultaneously improving patient outcomes and financial sustainability, SCSA supports both high-quality care and efficient resource utilization.

**Funding for the study:**

This study was funded by the manufacturer of SCSA.

